# The role of intraoperative frozen sections for thyroid nodules

**DOI:** 10.1016/S1808-8694(15)30787-4

**Published:** 2015-10-19

**Authors:** João Paulo Alves de Almeida, Sergio Dias do Couto Netto, Rafael Pinto da Rocha, Elio G. Pfuetzenreiter, Rogério Aparecido Dedivitis

**Affiliations:** 1 Medical Student - Faculdade de Ciências Médicas da Fundação Lusíada, Santos; 2 Medical Student - Faculdade de Ciências Médicas da Fundação Lusíada, Santos; 3 Medical Student - Faculdade de Ciências Médicas da Fundação Lusíada, Santos; 4 Head and Neck surgery resident - Hospital Ana Costa, Santos; 5 PhD in Medicine - Graduate Program in Otorhinolaryngology and Head and Neck Surgery - UNIFESP - Escola Paulista de Medicina

**Keywords:** fna, thyroid neoplasms, frozen sections, sensitivity and specificity, thyroidectomy

## Abstract

The role of intraoperative frozen sections (FS) during thyroidectomy is controversial.

**Aim:**

to evaluate the role of FS for thyroid nodules management.

**Patients and methods:**

All patients who had thyroid surgery for nodular disease and previous USG-guided FNAB in 2006 were prospectively analyzed. They underwent intraoperative FS evaluation, and the biopsy material was classified as benign, malignant or follicular neoplasm. FNAB, FS and paraffin sections were compared.

**Results:**

Under the FS, 54% of the nodules were benign, 30% were follicular neoplasms, and 16% were malignant. All cases considered benign and malignant under the FS evaluation were confirmed through the histological “paraffin” analysis. Since it is not considered a definitive indication for total thyroidectomy, if the follicular neoplasms were classified as “benign” under the FS, their sensitivity, specificity, positive and negative predictive values and global diagnostic accuracy were 69%, 100%, 100%, 91,5% e 77%, respectively. Among the 42 cases classified as “follicular neoplasm” under the FNAB, in 1 case the FS conclusion was for papillary carcinoma, in 3 cases as benign (all confirmed through the “paraffin”); and 38 cases continued as “follicular pattern”, being 29 follicular adenomas and 9 carcinomas through the “paraffin”.

**Conclusion:**

The FS is only indicated when the FNAB reports “follicular neoplasm”.

## INTRODUCTION

The fact that fine needle aspiration (FNA) is a highly accurate preoperative method to be used for the diagnosis of thyroid nodules in cancer detection^1^, and the so-called “follicular neoplasia” still is a dilema^2^. The value of the intra-operative frozen section (FS) remains controversial while its potential to help the surgeon decide between hemithyroidectomy or total thyroidectomy. The method can potentially avoid a second surgery to remove the contralateral lobe should the surgical specimen reveal malignancy in the histopathology included in paraffin and, alternatively, it can avoid an unnecessary total thyroidectomy which will cause the patient to have to replace levothyroxine forever and increase the chance of the patient developing hypoparathyroidism and damage to the recurrent laryngeal nerve^3^.

The goal of the present investigation is to assess the value of the frozen section regarding decision making when facing a nodular disease of the thyroid gland.

## MATERIALS AND METHODS

During the year of 2006, in a prospective study, 126 patients were consecutively submitted to thyroidectomy because of a thyroid nodular disease, and the nodules were previously assessed by guided FNA. All the patients were submitted to FNA performed by the same ultrasound operator and pathologist and the pathology interpretation was carried out by the same pathologist who participated in the harvesting of the material. During surgery, all the specimens were submitted to intraoperative frozen section test. The histopathology diagnosis of the material embedded in paraffin was available. The present study was approved by the Ethics in Research Committee of the local institution.

The FNA is conducted through a 20mL plastic syringe with a 21 gauge needle. Ultrasound was performed by means of a 10MHz probe and a minimum of three aspirations was normally used without local anesthesia. In case of mixed nodules, the liquid component was initially emptied, and the punction was repeated afterwards. The material collected was assessed by the pathologist, and the liquid was previously centrifuged. All the material collected was fixed in alcohol and dyed by Papanicolaou or HE. The frozen section exam was made with one or two representative sections of the area most likely to present capsular invasion.

The cytopathology specimens were classified as inconclusive, benign (colloid nodule, cyst or thyroiditis), malignant and suspected malignant (specimens which definition of malignancy could not be established, presenting a follicular pattern). The presence of monomorphic epithelial cells or slightly pleomorphic, frequently grouped in micro-follicles or in syncytial masses and showing nuclei with atypia or eosinophilic aspect of Hurthle cells, were all considered follicular pattern. As to the frozen sections, the surgical specimens were classified into inconclusive, benign, malignant and follicular pattern.

The frozen section was compared to the histopathology exam (paraffin), considered gold standard. True positive and true negative cases were defined with basis on the histopathology confirmation of the frozen section, of carcinoma or benign lesion, respectively. Thus, the disagreeing results were classified into false-positive and false-negative. Sensitivity, specificity, the predictive values of the negative and positive tests and the accuracy were calculated.

Following that, the FNA findings were compared to those from the frozen section and the impact of each one in the establishment of the surgical approach (partial or total thyroidectomy) was assessed.

## RESULTS

Comparing the FS with the paraffin histopathology (Gold Standard).

**Figure 1:**
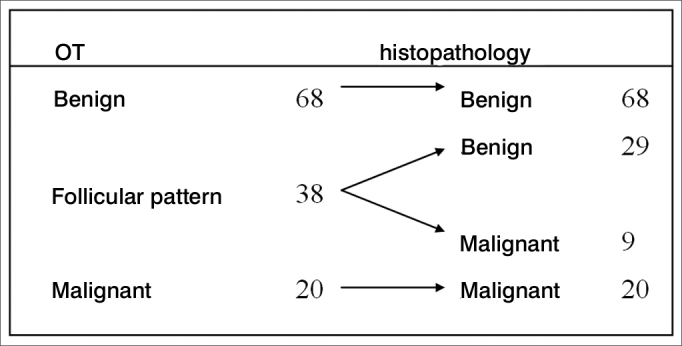
Comparing the FS and the paraffin findings.

In the present sample, there was no FS deemed inconclusive: 68 nodules (54%) were benign, 38 (30%) were follicular neoplasias (we must wait for the results of the paraffin study for a detailed investigation of vascular and capsular invasion) and 20 (16%) were malignant. Figure 1 compares the FS findings with the histopathology (“paraffin”).

Among the 20 cases considered malignant at the FS, two were thyroid medullary carcinomas and all the others were papilliferous carcinoma. Among the cases classified as follicular pattern (38), 29 were benign (follicular adenomas) and nine malignant, two cases of follicular carcinoma and seven of papilliferous carcinoma - follicular variant.

**Figure 2:**
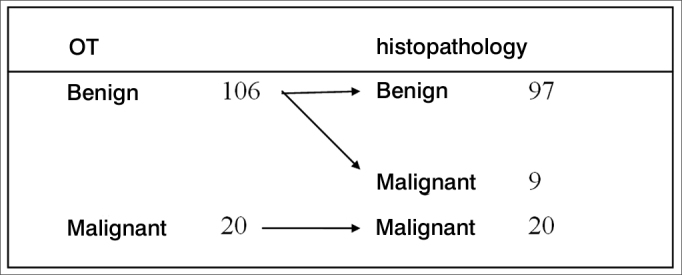
Comparing the FS and the paraffin findings, considering the suspicious punctions (follicular pattern) as being benign.

If we disregard the follicular pattern punctions, the predictive values for the negative (benign punction) and positive (malignant punction) tests add up to 100%. However, should the FS be suspicious (follicular pattern), it is one indication to perform total thyroidectomy, for the lack of criteria to conclude for malignancy - if classified as “benign”, we found a new status (Figure 2).

In this new status, considering the follicular pattern cases (wait for “paraffin” result) as “benign” in the FS, the following values were found: sensitivity = 69%; specificity = 100%; predictive value for the positive test = 100%; predictive value for the negative test = 91.5%; and accuracy = 77%.

### Comparing FS with FNA

All the cases had been submitted to FNA in the preoperative test. Thus, of the 126 nodules punctured, 65 (51.6%) were benign, 42 (33.3%) were follicular neoplasia and 19 (15.1%) were malignant. Crossing these data with those obtained from the FS exam, we noticed the following:

1) the 19 cases characterized as “malignant” by the FNA were confirmed by the FS and the paraffin;
2) the 65 cases characterized as benign in the FNA were confirmed by the FS and by the paraffin;
3) considering the 42 cases reported as follicular neoplasia by the FNA, we had:

– in one case, the FS found the criteria matching those of papilliferous carcinoma (confirmed by the paraffin test);
– in three cases, FS found enough criteria to define it as being benign (confirmed by the paraffin test);
– in the remaining 38 cases, the FS kept the appearance of a follicular pattern, suggesting that one should wait for the paraffin results; of these, 29 came as follicular adenomas and nine came as carcinoma, two follicular carcinoma and seven papilliferous of the follicular variant.

## DISCUSSION

Table 1 shows the study regarding the results obtained with the FNA on the assessment of thyroid nodules^4^, ^5^, ^6^, ^7^, ^8^, ^9^, ^10^, ^11^, ^12^, ^13^, ^14^, ^15^, ^16^, ^17^, ^18^, ^19^, ^20^, ^21^, ^22^, ^23^, ^24^, ^25^, ^26^, ^27^, ^28^, ^29^, ^30^, ^31^, ^32^, ^33^, ^34^, ^35^, ^36^, ^37^, ^38^, ^39^, ^40^, ^41^, ^42^, ^43^, ^44^, ^45^, ^46^, ^47^, ^48^.

**Table 1:** FS results in the assessment of thyroid nodules.

Author	n	Sensitivity	Specificity	PPV	PNV	Accuracy
Bugis et al., 1986^4^	198					95%
Shaha et al., 1990^5^	190					95%
Rosen et al., 1990^6^	457	53%	100%	100%	97,8%	97,9%
Shaha et al., 1990^7^	38					95%
Irish et al., 1992^8^	137					87%
Kingston et al., 1992^9^	395	52%	100%	100%	73%	79%
Gibbet al., 1995^10^	85					86%
McHenry et al., 1996^11^	76	93%	100%			97%
Godei et al., 1996^12^	2470	74%	100%			
Morosini et al., 1997^13^	812	91,3%	100%			97,4%
Paphavasit et al., 1997^14^	1023	78%	99%	90%	98%	98%
Chang et al., 1997^15^	586			97%	95,5%	92,6%
Linder et al., 1997^16^	73	83%		95%		
Mulcahy et al., 1998^17^	66					92%
Chen et al., 1998^18^	57	23%				
Hamming et al., 1998^19^	240	67%	99%	98%	87%	89%
Tworek et al., 1998^20^	68		98%			
Boyd et al., 1998^21^	151	86%	99%			96%
Ng SC et al., 1999^22^	34	100%	86%			
Chow et al., 1999^23^	84					100%
Multanen et al., 1999^24^	335	74,6%				
Taneri et al.2000^25^	63			28,5%	77,5%	
Piraino et al., 2000^26^	85	89,4%				
Lin et al., 2000^27^	63	87%				
Leteurtre et al., 2001^28^	63	17%				
Tamimi et al., 2001^29^	61	60%	100%			90%
Bastagli et al., 2001^30^	155	42,9%	100%	100%	8,5%	92%
Lee et al., 2002^31^	1076					90,5%
Abboud et al., 2003^32^	113	68%	99%			
Pisanu et al., 2003^33^	36	33,3%				
Boutin et al., 2003^34^	163	73%	99%			
Kesmodel et al., 2003^35^	42	36%				
Saydam et al., 2003^36^	67	100%	87%			91%
Callcut et al., 2004^37^	152	67%	100%	100%		96%
Lumachi et al., 2004^38^	606	83%	100%			97%
Cetin et al., 2004^39^	203	87,1%	100%			97,8%
Rios et al., 2004^40^	197	19%	100%	100%	93%	93%
Pisanu et al., 2004^41^	41	33,3%				
Furlan et al., 2004^42^		56,1%				
Sahin et al., 2005^43^		84%	100%			
Chao et al., 2005^44^	135	40%	100%	100%	92%	92,9%
Dzodic et al., 2006^45^	40	77,7%	100%	100%	94%	95%
Giuliani et al., 2006^46^	417	56,25%	98,16%	81,81%	93,85%	
Olson et al., 2006^47^	236	25%				
Miller et al., 2007^48^	205	23%	99%			78%

Our findings match those in the literature, with good accuracy, nonetheless, it also fails when compared to the so called “follicular pattern”. Thus, specificity and positive predictive value are high. We found 100% for both, matching a good part of the data in the literature. This means that, when the FS method points to a cancer possibility, such result is highly reliable. The “follicular pattern” results come with the pathologist's recommendation of waiting for the “paraffin” result, because the criteria necessary for the final diagnosis of malignancy were not found, thus not systematically recommending total thyroidectomy. With this, on the 2×2 Table such conclusion was classified as “benign” and this justifies the 69% sensitivity in our sample. Now, when the FNA is considered, the finding of “follicular neoplasia” is a criterion for surgical indication, thus, it must be classified as “malignant”^1^.

There was a strong correlation between the benign and malignant findings among the FNA guided by ultrasound, FS and histopathology, embedded in “paraffin” (Gold Standard). Thus, when the FNA shows it is benign, or malignant, the FS did not add information. Now, within the 42 cases of “follicular neoplasia” seen at the FNA, in a FS found malignancy criteria, with an impact on the treatment decision and, in three, it was defined that it was a benign lesion.

## CONCLUSION

The FS is only indicated in cases which the FNA yielded results of “follicular neoplasia”.
